# Performance optimization of In(Ga)As quantum dot intermediate band solar cells

**DOI:** 10.1186/s11671-023-03839-z

**Published:** 2023-04-20

**Authors:** Guiqiang Yang, Wen Liu, Yidi Bao, Xiaoling Chen, Chunxue Ji, Bo Wei, Fuhua Yang, Xiaodong Wang

**Affiliations:** 1 Engineering Research Center for Semiconductor Integrated Technology, Institute of Semiconductors, Chinese Academy of Sciences, Beijing, 100083 China; 2 Center of Materials Science and Optoelectronics Engineering, University of Chinese Academy of Sciences, Beijing, 100049 China; 3 School of Integrated Circuits, University of Chinese Academy of Sciences, Beijing, 100049 China; 4 Beijing Engineering Research Center of Semiconductor Micro-Nano Integrated Technology, Beijing, 100083 China

**Keywords:** In(Ga)As quantum dot, Intermediate band solar cell, Strain, Thermal excitation, Carrier lifetime

## Abstract

Quantum dot intermediate band solar cell (QD-IBSC) has high efficiency theoretically. It can absorb photons with energy lower than the bandgap of the semiconductor through the half-filled intermediate band, extending the absorption spectrum of the cell. However, issues in the IBSC, such as the strain around multi-stacking QDs, low thermal excitation energy, and short carrier lifetime, lead to its low conversion efficiency. In recent years, many efforts have been made from different aspects. In this paper, we focus on In(Ga)As QD-IBSC, list the experimental technologies used to improve the performance of the cell and review the recent research progress. By analyzing the effects of different technologies on conversion efficiency, the development direction of the In(Ga)As QD-IBSC in the future is proposed.

## Introduction

Sunlight has a spectral range of about 0.5–4 eV, but the traditional solar cells with a single bandgap can only utilize the short wave and the long part is completely wasted. According to a theoretical calculation, the energy conversion efficiency limitation of the single-junction solar cell, which was determined by Shockley and Queisser (SQ limit), is only 40.7% under full concentration light [1]. There are two main reasons why the cells can’t be more efficient. On the one hand, photons with energies lower than the band gap are not absorbed, making this part of energy unavailable. On the other hand, for photons absorbed, only the portion of the energy larger than the band gap of matrix is converted, and the other is wasted as heat [2]. To address the issue and increase the conversion efficiency of solar cells, the IBSC was proposed by A. Luque and A. Marti in 1997 [3]. It can absorb photons with energy lower than the band gap of the matrix through the IB and increases the short-circuit current density (*J*_sc_) of the cells with open-circuit voltage (*V*_oc_) preserved, since the *V*_oc_ is limited by the matrix band gap [4, 5]. The maximum conversion efficiency limit of the IBSC reaches 63.2% [6, 7]. At present, there are three main methods to realize the IB, including QDs, highly mismatched alloys, and bulk materials with deep-level impurities [8]. Among them, the QD-IBSC has received a lot of attention from researchers because it is easy to modify the band structure of the cell by adjusting size and density of QDs. Two conditions must be met to form a QD-IBSC: firstly, the IB must be half-filling to ensure that it can provide holes to the valence band (VB) and electrons to the conduction band (CB) [9]. Secondly, the CB, VB, and IB all have their independent quasi-Fermi levels (QFL), and only in this way will the maximum voltage be limited by the band gap of semiconductors [10, 11].

So far, many materials have been used as substrates for the cells, such as Si, InGaP, GaAs, and so on. For example, research on Si-based cells with Ge QDs shows that the absorption in the infrared region is enhanced, and the *J*_sc_ increases [12]. However, the QDs lead to the decrease in the effective band gap and a drop in *V*_oc_. More research focuses on III–V compound semiconductors at present, like InGaP, GaAs, etc., since most of them are direct bandgap semiconductors with strong light absorption, good radiation resistance, and low temperature coefficient. For instance, InGaP has a wide band gap, which means that a large energy difference can be obtained between the confined state in QDs and the CB [13, 14]. For InP/InGaP QD-IBSC, the thermal excitation energy is about 0.35 eV and it has a long carrier lifetime of 30 ns, which is more conducive to implement the two-step photon absorption process [15]. In(Ga)As/GaAs QDs based on S-K growth mode have been widely studied in semiconductor lasers, detectors, and other aspects [16–19], and the growing technology and material properties have been well mastered [20–23]. Therefore, In(Ga)As/GaAs are suitable to study the properties and performance of QD-IBSC. At present, much attention has been taken to the well-known system In(Ga)As/GaAs. And experimental results proved that there exists the two-step absorption process and the *J*_sc_ of the cell with QDs increases [24, 25]. Although it has been proved that it is feasible to absorb low energy photons through the IB and different kinds of QD-IBSCs have been tried, the results are less than satisfactory. The transition rate between the IB and the CB (~ 10^3^ s^−1^) is typically less than that between the VB and the IB (~ 10^9^ s^−1^) due to small solar cell power density, optical absorption cross section and density of QDs [26]. The energy difference between the IB and the CB is small, which results in serious thermal excitation at room temperature, so it not only reduces the absorption of long-wavelength photons, but also leads to the thermal coupling of the IB and the CB and the drop of the *V*_oc_. Research shows that it can be reduced by low temperatures and concentrated light [27]. There exists strain around QDs grown through Stranski–Krastanov (S–K) mode because of the lattice mismatch between the matrix and the QDs, which limits the growth of multiple layers of QDs. These are the two common problems in QD-IBSC. In addition to these, there are also problems like half-filling, short carrier lifetime and so on [28–31]. To solve these problems, researchers have made efforts from different aspects.

In this paper, we explain the principle of the IBSC and the process of thermal excitation and non-radiation recombination. Then, various technologies are introduced to solve the problems mentioned above, like Si doping, cap layer, size control, etc. Finally, we summarize the technologies investigated and the future development direction of QD-IBSC is proposed.

## Principle of QD-IBSC

**Fig. 1:**
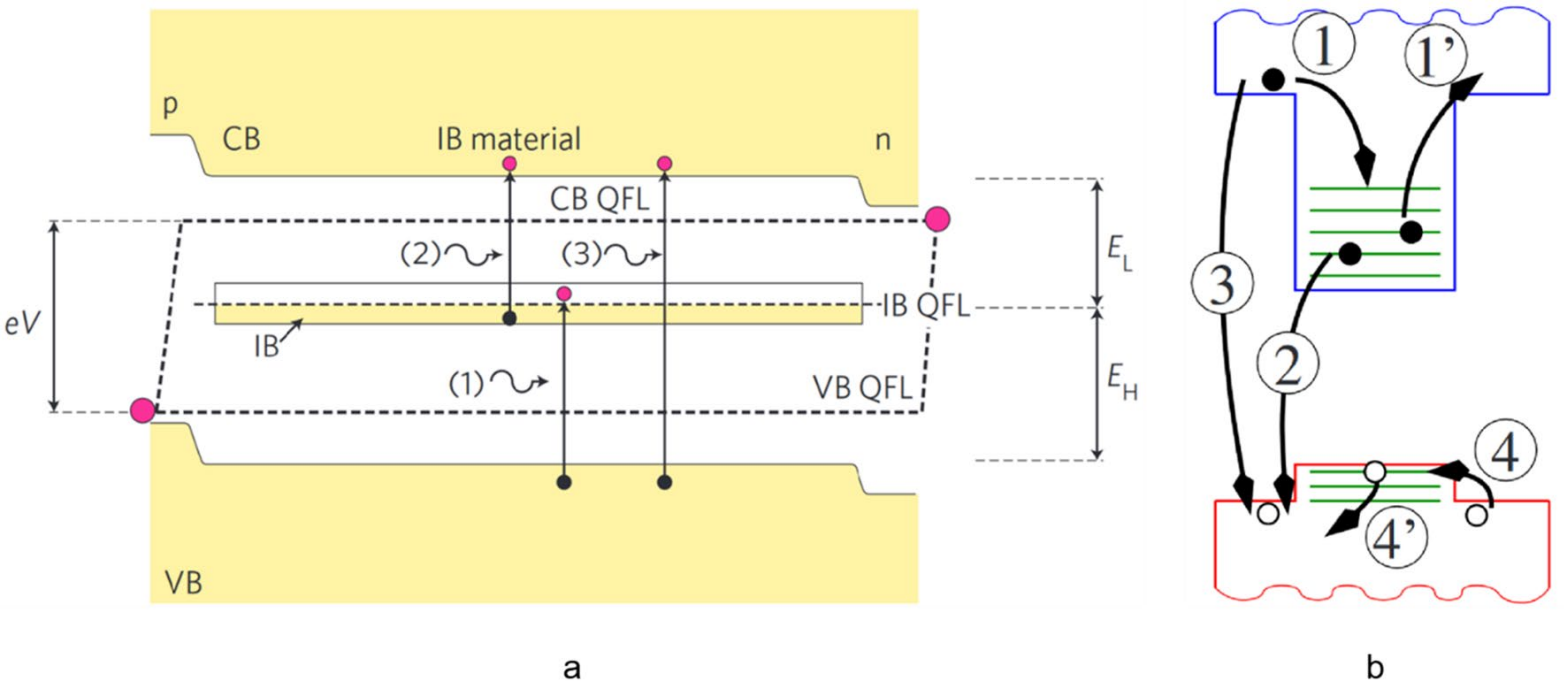
**a** Band alignment diagram of the IBSC. Reproduced from Ref. [32]. **b** the transition of electrons from the CB to the IB (process1); the thermal electron escape from the IB electrons to the CB (process 1’); the recombination of the IB electrons and the VB holes (process 2); the recombination of the CB electrons and VB holes (process 3); the transition of hole from the VB to the dot (process 4); the thermal hole escape from dot to the VB (process 4’). Reproduced from Ref. [33]

The band alignment of the QD-IBSC is shown in Fig. 1a. The QD states form the IB in the substrate band gap, which allows two ways for electrons to transfer from the VB to the CB. One is for the material to absorb photons, and the electrons in the VB are directly transferred to the CB; the other way is for the electrons to absorb sub-bandgap energy photons via transitions from the VB to the IB and then the IB to the CB. By adding an IB between the VB and the CB, the cells are able to absorb photons with low energy via a two-photon absorption process, and the absorption spectrum is expanded, thus improving the conversion efficiency. To achieve the absorption process, the IB must be located at a suitable position to prevent thermal carriers escape and tunneling, and half-filled to ensure that it facilitates carriers to transfer between the VB and the CB. The optimal bandgap for the IBSC is 1.95 eV, which is split by the IB into two sub-bandgaps of approximately 0.71 eV and 1.24 eV [32].

The efficiency of solar cells is as follows:

1 $$\eta = \frac{{{\text{FFV}}_{{{\text{oc}}}} J_{{{\text{sc}}}} }}{{{\text{Pin}}}}$$

where *P*_in_ is the input power, *V*_oc_ is the open-circuit voltage, *J*_sc_ is the short-circuit current density and FF is the fill factor. Therefore, to improve its efficiency, *J*_sc_ should be increased as much as possible while the *V*_oc_ is preserved [34].

Figure 1b shows various excitation and recombination processes in the IBSC [33]. To obtain high efficiency, it is necessary to prevent thermal carriers escape and recombination processes in IBSCs. If the three QFLs separate from each other, the *V*_oc_ will only be limited by the band gap of the matrix. However, there exists thermal escape which is related to the thermal coupling of the IB and the CB, and the electrons in IB transfer to the CB through the thermal excitation rather than the second-photon absorption, resulting in the drop of *V*_oc_. And the excited states of QDs and the confined states of wetting layer will assist the process, which should be avoided as much as possible [35, 36]. For recombination in solar cells, they can be radiative recombination and non-radiative recombination. Radiation recombination is caused by a detailed balance counterpart of the light absorption process, which is inevitable. For non-radiative recombination, it can be Auger recombination transferring the energy to other carriers or SRH recombination to phonons. Auger recombination occurs in highly doped semiconductors, so it is not important in less doped materials. The generally accepted cause of SRH recombination is the lattice relaxation multiple-phonon emission mechanism (MPE). Research shows that SRH recombination can be inhibited by increasing the density of impurities. And this prevents the IB to act as recombination centers [37, 38].

## Research progress of In(Ga)As/GaAs QD-IBSC

### Doping of Si

**Fig. 2:**
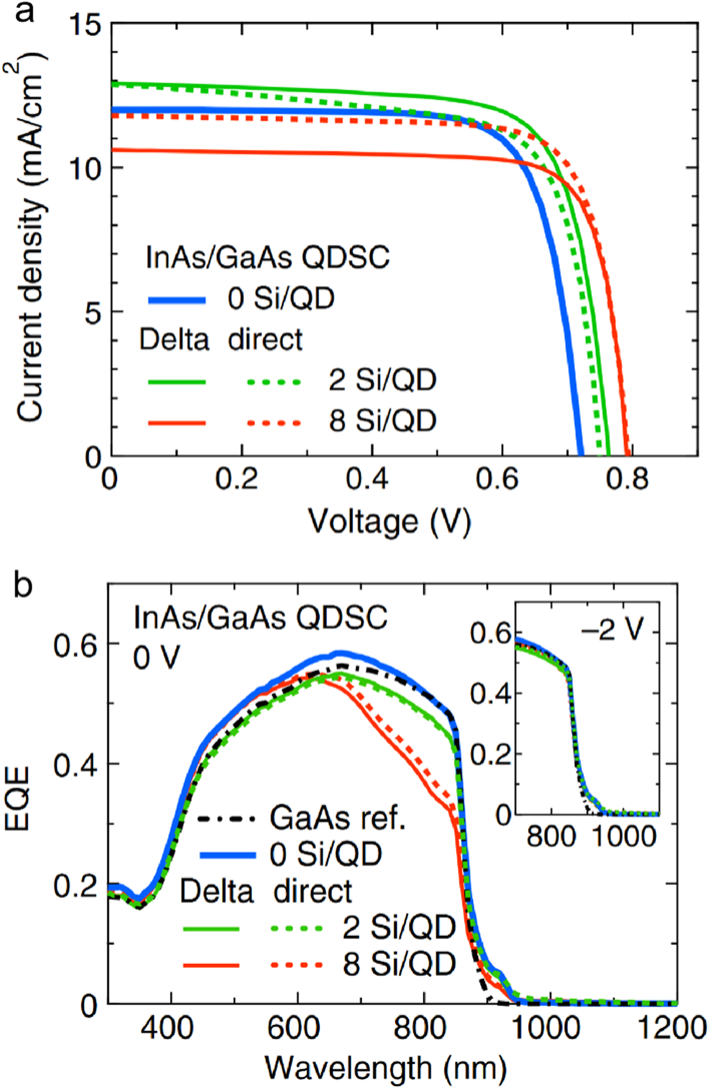
**a** EQE characteristics of InAs/GaAs with *δ* doping (solid line) or direct doping (dashed line). **b** J-V characteristics of InAs/GaAs with*δ*doping (solid line) or direct doping (dashed line). Reproduced from Ref. [40]

Half-filling of the IB is important in QD-IBSCs, which allows the absorption of sub-bandgap photons by the two-step photon absorption. It is proved that the half-filling can be achieved by Si doping, which has little influence on the splitting of the QFLs [39]. There are two doping methods: *δ* doping and direct doping, both of which have been observed larger thermal excitation energy and improvement in *J*_sc_ or *V*_oc_ (in Fig. 2), it is thought that the doping has an effect on the optical processes and inhibits the non-radiative recombination. And for the heavy doping QDs, the drop in current is attributed to the increased rate of electron capture into QDs since the energy band of QD region becomes more flattened [40, 41]. The Si dopants incorporated in wetting layer supply electrons to QDs, and a potential barrier is formed at the interface of positively charged wetting layer and negatively charged QDs. The barrier suppresses thermal escape from QDs and thus the *V*_oc_ recovers [42]. Morioka, Takayuki et al. found that recombination process in the QDs region was reduced due to the half-filled IB, leading enhancement in *J*_sc_, *V*_oc_ and conversion efficiency [43]. Yang, Xiaoguang et al. fabricated an InAs/GaAs QD-IBSC with the Si doping concentration of 5 × 10^11^ cm^−2^ and the conversion efficiency reaches 17% [44].

### Size of QDs

**Fig. 3:**
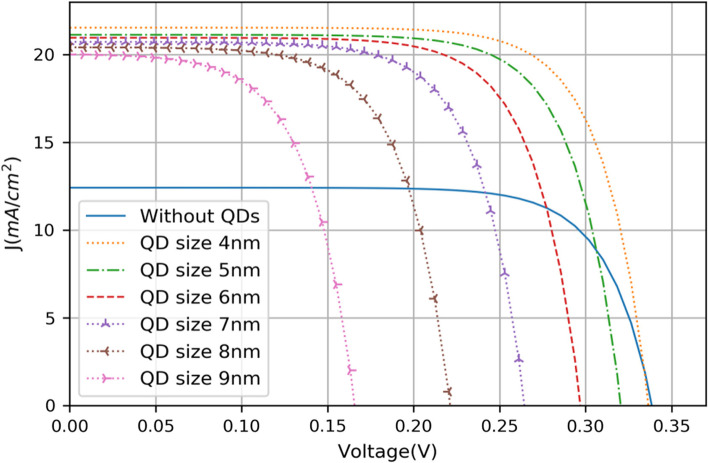
J–V characteristics of the InAs/GaAs with various sizes of QDs. Reproduced from Ref. [47]

The size is an important parameter to consider when growing QDs. The large QDs increase the absorption coefficient and extend the absorption spectra, while the IB is not formed in the band gap of the matrix [45, 46]. Therefore, it is necessary to optimize the size of QDs to obtain a confined electron level in the CB offset, namely the IB. As is shown in Fig. 3, the small QDs allow few excited states inside the CB offset, which is conducive to the splitting of QFLs between the IB and the CB, as well as a better position of the IB, so the thermal excitation energy increases and the voltage is preserved [36, 47, 48]. Also, the reduction of the QD width means that a higher QD density can be obtained, resulting in an increase in photocurrent [49]. For the InAs/GaAs QD-IBSC, it is calculated that the optimal QD size and distance between two QDs are 6–12 nm and 2 nm, respectively [45, 46]. And a low QD size dispersion prevents different confined energy levels to appear, but it may be hard to realize in QD-IBSC operation [50].

### Cap layer

**Fig. 4:**
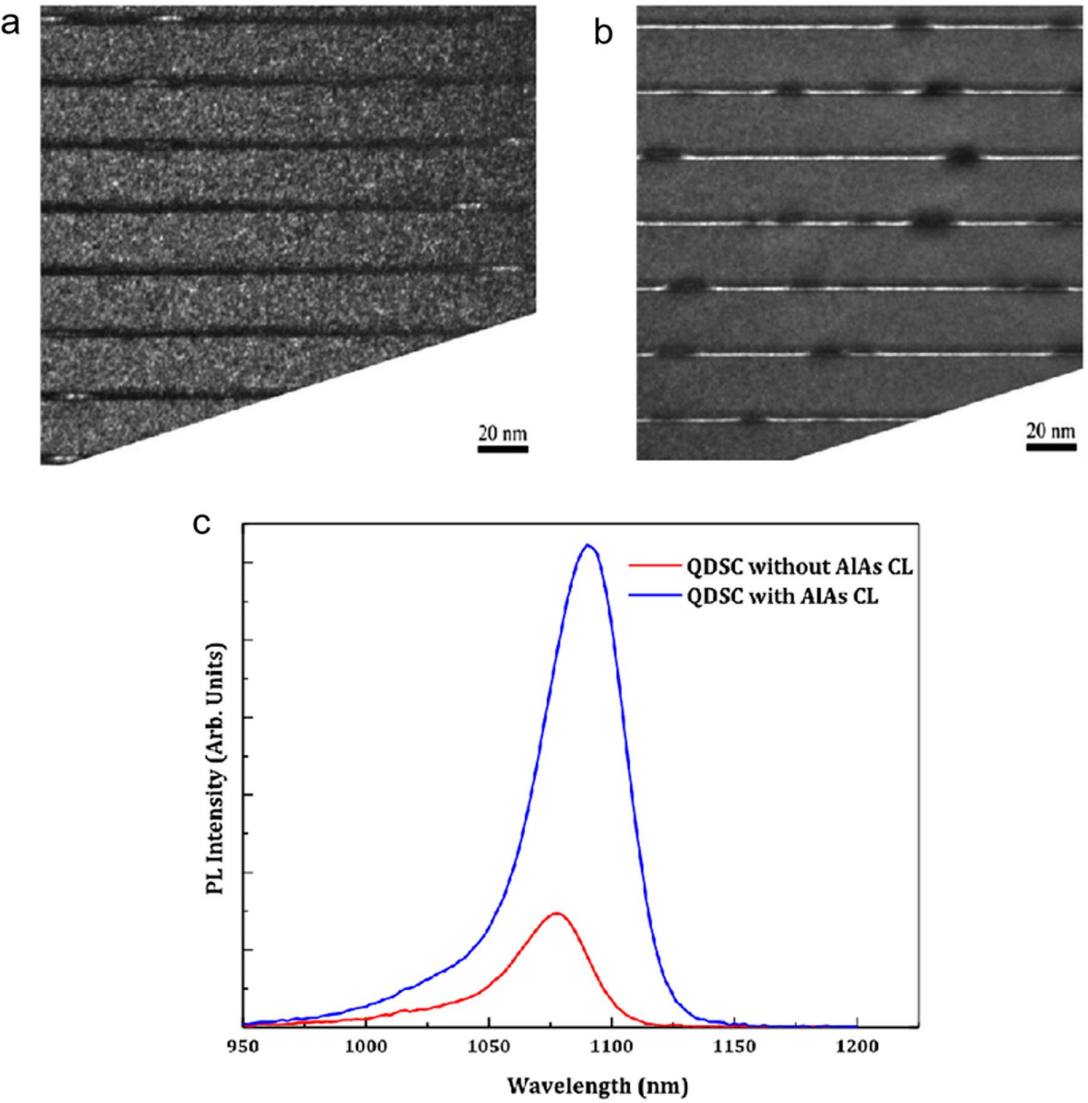
**a** TEM image of the InAs/GaAs without AlAs CL. **b** TEM image of the InAs/GaAs with AlAs CL. **c** PL spectra of the InAs/GaAs without (red) and with (blue) AlAs CL. Reproduced from Ref. [51]

Generally, QDs are grown by S-K mode, which will inevitably produce the wetting layer (WL), and the WL forms continuous confined energy levels below the CB, these energy states assist the thermal excitation of carriers and suppress the second-photon absorption process, leading to the connection of the IB and the CB and a drop of *V*_oc_ [51]. It is proved that the thin AlAs cap layer (CL) on InAs/GaAs QDs could suppress the formation of the WL and increase the PL intensity (Fig. 4), because the high intermixing between CL and WL results in an InAlGaAs layer with gradual composition [51–53]. Also, AlAs CL prevents in segregation and In-Ga intermixing, and reduces QDs decomposition [54, 55]. In addition to AlAs, the CLs commonly used include InGaAs, InAlGaAs, AlGaAsSb and so on, and different CLs have different effects [56–58]. Wei-Sheng Liu et al. fabricated an InGaAs/GaAs(Sb) QDs with AlGaAsSb CL. It showed an enlarged band gap with the inserting of AlGaAsSb CL and the *V*_oc_ is increased from 0.67 to 0.7 V [58]. T. Sugaya et al. found that the In_0.2_Ga_0.8_As CLs on In_0.4_Ga_0.6_As QD solar cells could reduce the strain around the QDs and 50-stack InGaAs QDs can be grown without defects. The absorption spectra are extended and the conversion efficiency of 10-stack InGaAs QDs reaches 12.2% [56].

### Strain compensation layer

In general, to improve the conversion efficiency, the researchers increase the layers of QDs to realize the high density of QDs. However, there exists a lattice mismatch between the matrix and the QDs. As the QD layers increase, strain will accumulate in the QDs, which will eventually lead to the generation of dislocations [59]. The dislocations clustered in the QDs propagate toward the surface of the cells, and the minority carrier lifetime decreases [60]. In order to solve the problem, strain compensation (SC) is carried out, in the case of the band gap and the lattice constant being considered in the selection of the SC layer. The band gap of the SC layers should not be much smaller than that of the matrix, but it should be similar to that of the matrix [61]. For InAs/GaAs, the layers doped with N, P, and Sb are generally used [29, 62, 63]. For example, no dislocations or coalesced islands were observed in 50-stack InAs QDs with GaNAs SC layers, a high density of QDs was achieved and the *J*_sc_ increased without a drop of *V*_oc_ [64]. S. M. Hubbard et al. used GaP layers to offset compressive strain in stacked InAs QDs and found that without the GaP tensile SC layers, the conversion efficiency of InAs/GaAs QD-IBSC decreased due to the strain-induced dislocations. While there are GaP layers, the conversion efficiency was increased from 3.7 to 10.8%, which indicated that the SC layers result in a reduction of the recombination process [65]. As a surfactant, antimony (Sb) reduces the surface energy, which in turn increases the density of QDs and reduces the coalescence dots [66]. Liu et al. [63] studied ten layers of InAs QDs, and each layer of QDs was grown with a GaAsSb SC layer. They found that the size of QDs became more uniform, and the *J*_sc_ was enhanced by 8.8% compared to that without GaAsSb, because it enhanced the absorption of the QDs around 1200 nm.

**Fig. 5:**
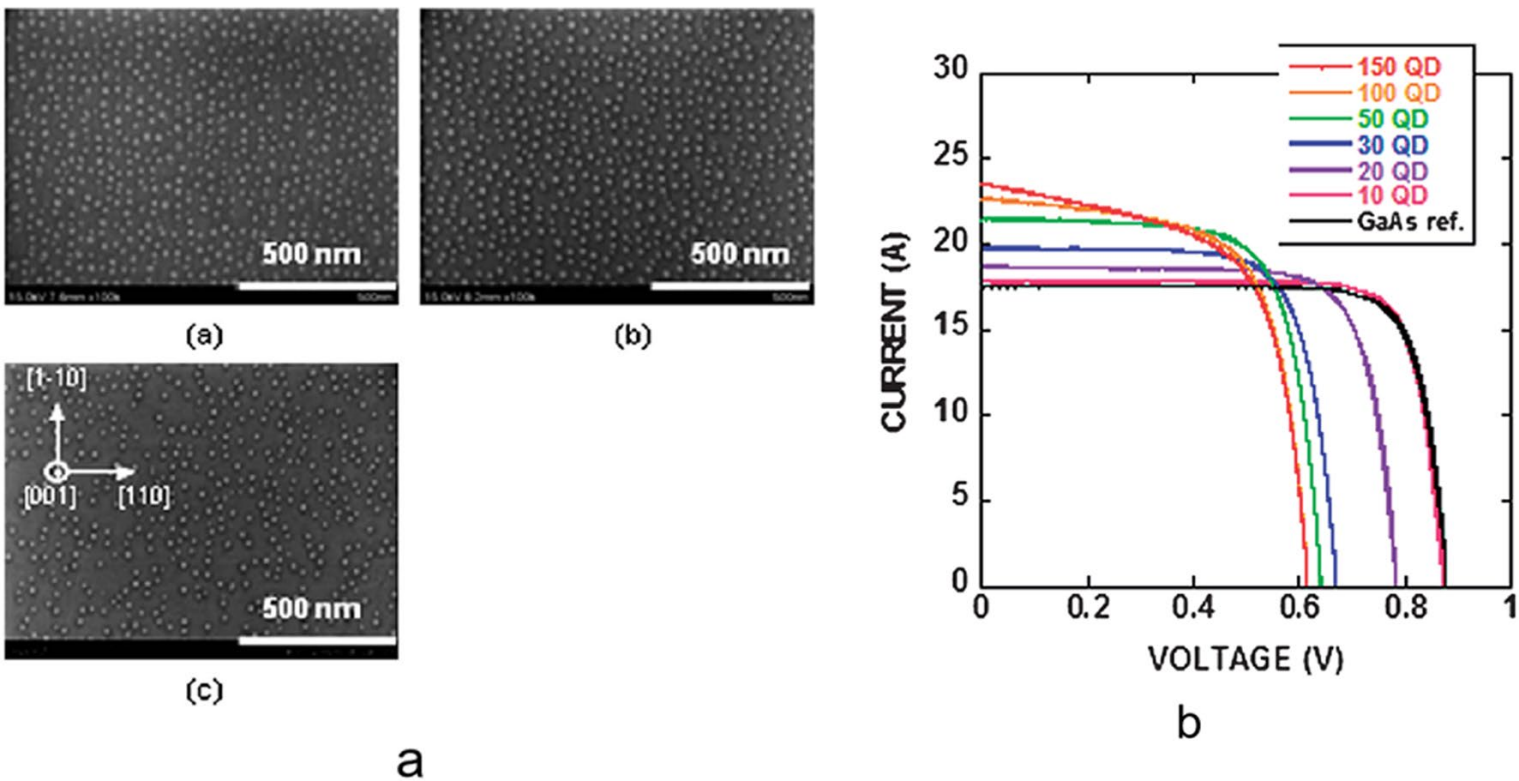
**a** SEM images of the surface plane on top of **a** 200, **b** 300, **c** 400-stack In_0.4_ Ga_0.6_As QD structures. **b** I–V characteristics of In_0.4_Ga_0.6_As/GaAs QD-IBSCs with different QD layers and a GaAs reference cell. Reproduced from Ref. [67]

In addition to the use of SC layers, the composition of the matrix or the QDs can be adjusted to reduce the lattice mismatch and the strain around the QDs. Voicu Popescu et al. fabricated an In_*y*_Ga_1-*y*_As/GaAs_1–*x*_P_*x*_ QD-IBSC, and the results showed a good lattice match, and the calculations indicated that a maximum thickness of 6 ML could be grown [33]. As shown in Fig. 5, the In_0.4_Ga_0.6_As/GaAs structure grown by Sugaya, Takeyoshi et al. was able to grow 400 layers without defects, but the *V*_oc_ decreases with the increasing of stacking QD layers, which is attributed to the recombination in QDs [67].

### Increase matrix band gap

**Fig. 6:**
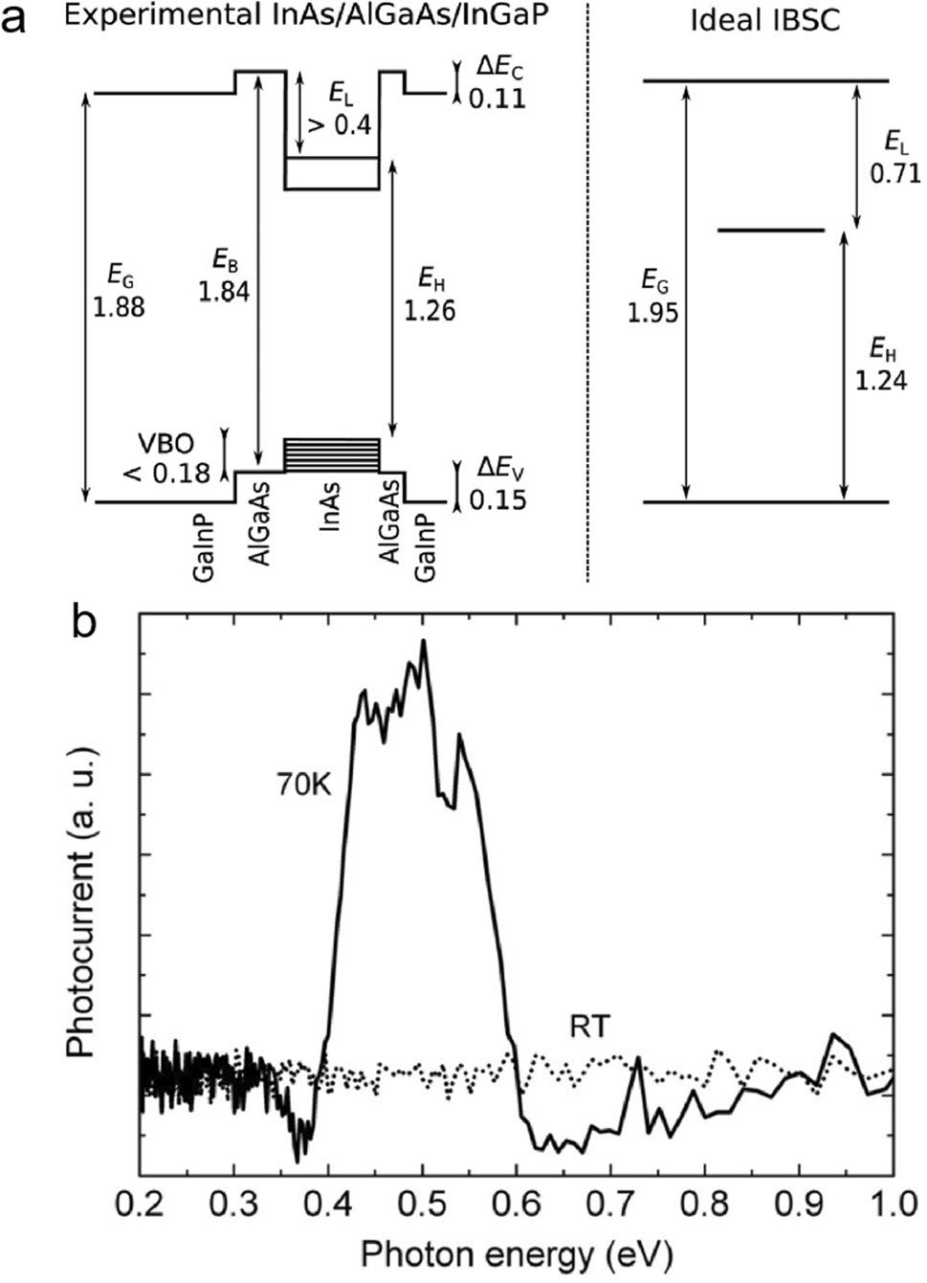
**a** Band structure diagram of the InAs/AlGaAs/InGaP (left) and an ideal QD-IBSC (right). **b** Photocurrent response to mid-infrared illumination of InAs/AlGaAs/InGaP at RT (dotted line) and 70 K (solid line). Reproduced from Ref. [73]

Although InAs/GaAs has been studied the most, it is not optimal for the IBSCs, because its effective band gap of 1.066 eV is less than the optimal value of 1.95 eV. Moreover, small QDs are necessary to make sure that the confined states separate from each other, but it will cause the first electron confined state to appear at high energy level. As a consequence, the energy difference between the IB and the CB is usually less than 0.2 eV, and there exists thermal carrier escape from the IB to the CB at room temperature, which results in a drop of the *V*_oc_ [68]. The theoretical calculation shows that the efficiency limit is only 49.44% for InAs/GaAs QD-IBSC by optimizing the QD size [36]. Thermal excitation energy can be increased by using the wide bandgap substrates, changing the GaAs barrier to AlGaAs, InGaP or GaAsP [69–71]. Ramiro et al. [72] increased the band gap of the matrix by doping Al into GaAs and showed that the thermal excitation energy reached 361 meV when the Al content reached 25%, which indicates that, if the energy split between the CB and the IB is large enough, the gap will suppress thermal carrier escape at room temperature. In addition to this, InAs/InGaP QD-IBSC prototypes with a wide bandgap were fabricated by them (Fig. 6), which have a band gap of 1.88 eV, and the energy difference between the IB and the CB is above 0.4 eV, leading to large thermal excitation energy [73].

### Type-II IBSC

**Fig. 7:**
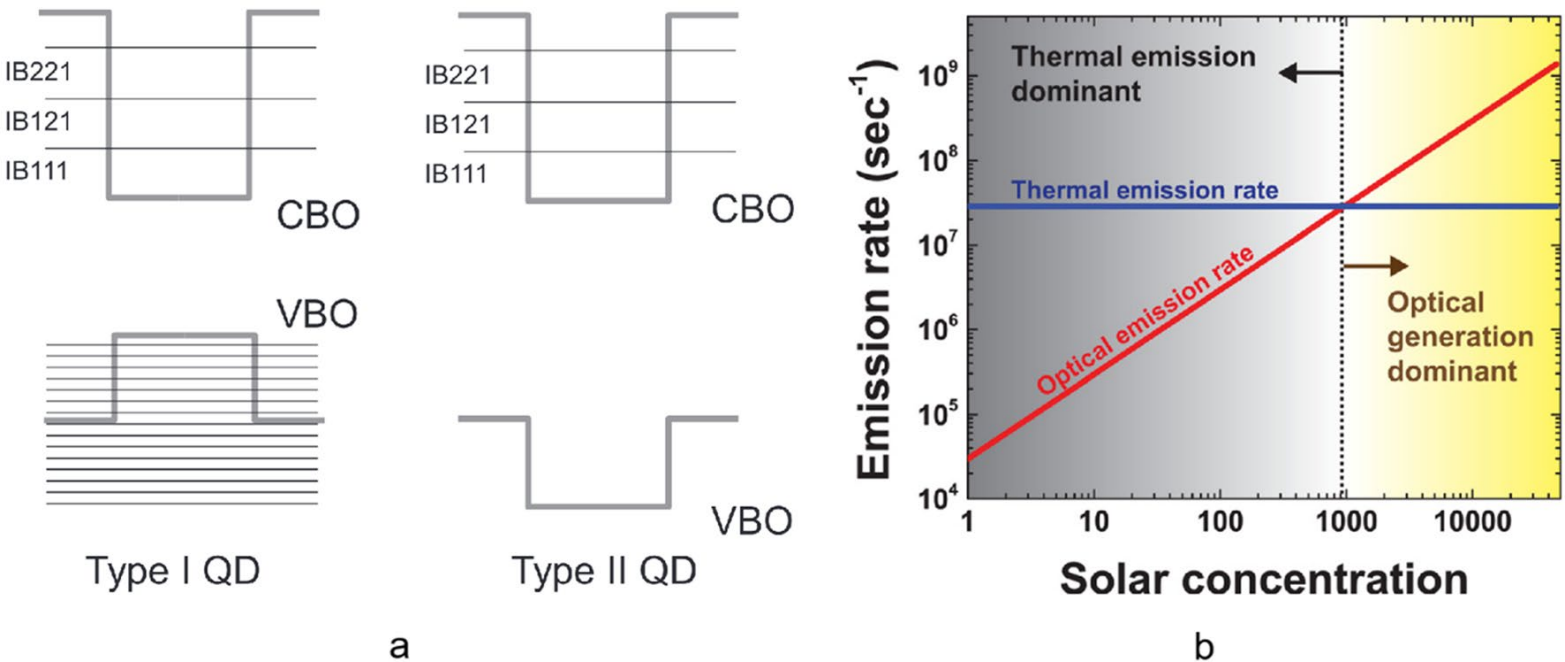
**a** Band diagram of type-I and type-II QD-IBSCs. Reproduced from Ref. [74]. **b** Optical (red) and thermal (blue) emission rates of holes in GaSb/GaAs QDs under different solar concentration. Reproduced from Ref. [83]

As shown in Fig. 7a (left), the CB and the VB of QDs are located in the bandgap of the substrate in the type-I QD-IBSC. The effective mass of the heavy holes is large, resulting in a tight arrangement of confined states of QDs around the VB and reducing the effective bandgap [74]. And if the energy levels of the IB and the CB are close, the thermal excitation will dominate the transition from the IB to the CB, which is harmful for the preservation of the *V*_oc_. The efficient radiative recombination in type-I IBSC leads to short carrier lifetimes (~ 1 ns) and a small number of photocarriers in QDs, reducing the two-photon absorption process since the second-photon absorption is related to the photocarrier in the IB [75–77]. Besides, the huge difference in the two optical transition rates associated with the IB is unfavorable for efficient QD-IBSCs [78]. Although the thermal excitation process can be suppressed by low temperature and concentrated light (Fig. 7b), it is not very realistic in practice [79]. To reduce these effects structurally, A. Marti and A. Luque proposed a type-II IBSC prototype with the band structure shown in Fig. 7a (right). The electrons and holes are spatially separated, so the carrier recombination in QDs is suppressed and the carrier lifetime increases [80]. As distinct from the type-I QDs, the bands of the type-II QDs and the substrate are staggered, so electrons and holes are spatially separated [74, 81]. And the type-II QD-IBSCs have higher thermal excitation energy than the InAs/GaAs, and this suppresses thermal carrier escape in QDs [82]. Besides, the hole confined states of QDs are located in the VB, so it prevents the reduction in the effective band gap.

**Fig. 8:**
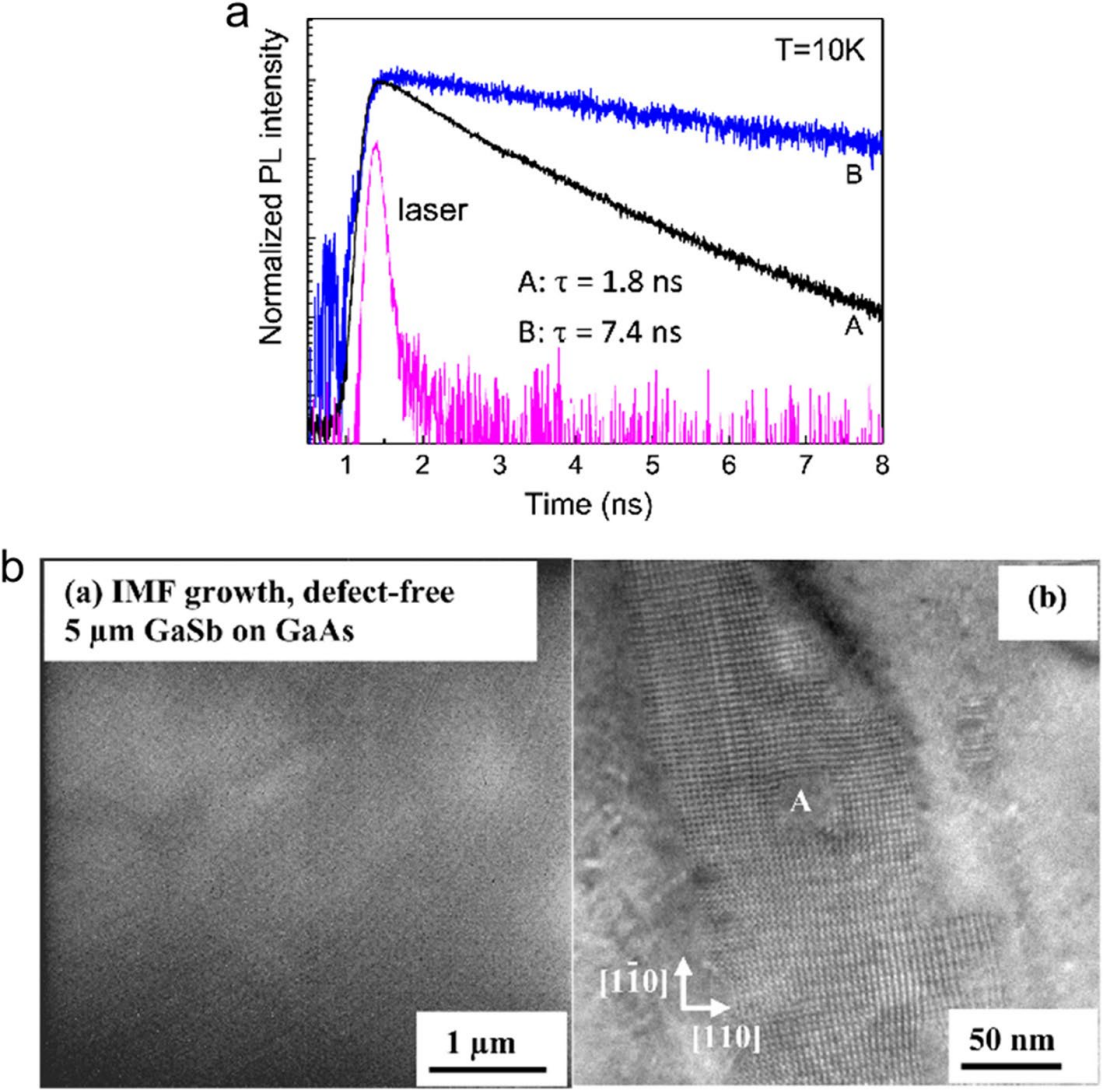
**a** PL intensity of sample A (InAs/GaAs_0.89_Sb_0.11_) and sample B (InAs/GaAs_0.85_Sb_0.15_) measured at *T* = 10 K. Reproduced from ref. [87]. **b** a is the TEM image of region free of dislocations and b is the TEM image showing the IMF formation and the gradual mergence process. Reproduced from Ref. [89]

A theoretical calculation shows that the efficiency of type-II QD-IBSC formed by InAs/GaAsSb can reach 18.4%, which exceeds the efficiency of the InAs/GaAs IBSC (17.8%) [84]. Whether the InAs/GaAsSb forms the type-I or the type-II depends on the component content of Sb in the GaAsSb layer [85]. Hatch et al. [86] found that InAs/GaAsSb showed type-II behavior when the Sb composition reached 12%. As shown in Fig. 8a, with the increase in Sb component, the carrier lifetime increases from 1.8 to 7.4 ns, which is attributed to the formation of the type-II band alignment [87]. In order to reduce the wave function overlap of the electrons and holes as well as prolong the carrier lifetime, a 2-nm GaAs wall is inserted between QDs and GaAsSb layers, and the carrier lifetime reached 480 ns [75]. It is noted that it will prevent the holes from tunneling when the GaAs wall is too thick, leading to carrier recombination in QDs and short carrier lifetime [88].

In addition to In(Ga)As QD-IBSC, GaSb/GaAs is another kind of type-II QD-IBSC, and the GaSb QDs can be grown by the interfacial misfit (IMF) dislocation array growth mode. The misfit array is located at the interface of GaSb/GaAs and is comprised of two 90° dislocations, and the strain energy is relieved by the periodic array misfit dislocations, so there are no visible defects can be observed on the GaSb surface (Fig. 8b)which is conducive to realize high-density QDs and enhances light absorption [89, 90]. GaSb/GaAs QD-IBSC confines holes only, and the IB is 0.49 eV above the VB, larger than energy difference between the IB and the CB of InAs/GaAs [91]. However, the experimental results show that the *J*_sc_ increases, but the *V*_oc_ is still decreased compared with that without QDs, attributing to the IB-VB thermal connection. Nevertheless, there is evidence shows that the half-filling of the IB is beneficial to the two-photon absorption process [83, 92].

From the above analysis, it can be concluded that different technologies have different impact on the performance of the QD-IBSC. The half-filling of the IB can be realized by Si doping; QD-IBSCs with suitable quantum dot size have higher output voltage and photocurrent; the CL suppresses the formation of the wetting layer, so the voltage is preserved; the SC layer reduces the strain around the quantum dots, which means more quantum dots can be grown and the light absorption is enhanced; increasing matrix band gap can reduce the effect of carrier thermal escape; long carrier lifetime can be obtained by the use of type-II quantum dots. Therefore, the effects of each technology must be taken into account in order to obtain high efficiency QD-IBSCs.

## The experimental record summary of QD-IBSCs

In this section, we present Table 1, which includes the various experimental results such as the conversion efficiency, *V*_oc_, *J*_sc_, and so on. If reported experimental evidence exists, the data are referenced. An oblique line case appears otherwise.

**Table 1:** Experimental record summary table of QD-IBSCs

Types of QD	Numbers of QD layers	Density of QDs/cm^−2^	*V*_oc_/V	*J*_sc_/mA cm^−2^	FF/%	*η*/%	Technologies	References
InAs/GaAs	5	4.2 × 10^10^	0.87	18.4	81.2	13	/	[99]
InAs/GaAs	5	3.2 × 10^10^	0.84	27.6	73.49	17	Direct Si-doping	[44]
InAs/GaAs	20	4.8 × 10^10^	0.79	6.5	∕	3.9	AlAs CL	[52]
InAs/GaAs	5	5 × 10^10^	0.83	23.9	77	10.8	GaP SC layer	[65]
In_0.4_Ga_0.6_As/GaAs	10	∕	0.868	17.7	80.5	12.4	/	[67]
InAs/GaN_0.01_As_0.99_	20	5.0 × 10^10^	0.545	32.95	65.9	11.82	Direct Si-doping	[43]
InAs/GaAs	20	3.0 × 10^10^	0.882	14.7	73.2	9.5	Direct Si-doping	[42]
InAs/GaAs	20	1.2 × 10^10^	0.78	24.3	72	14.0	*δ* Si-doping	[100]
InAs/GaAs	20	1.1 × 10^11^	0.71	8.3	∕	4.1	Sb-mediated growth	[101]
GaSb/In_0.15_Ga_0.85_As	5	8.7 × 10^9^	0.56	13.23	66.6	4.89	/	[102]
InAs/GaAs_0.86_Sb_0.14_	20	4.3 × 10^10^	0.70	18.0	71.3	9.0	GaAs interlayer	[75]
InP/InGaP	10	/	1.135	13.9	80.8	12.8	Wide bandgap matrix	[103]
In_0.4_Ga_0.6_As/GaAs	20	6.5 × 10^10^	0.747	19.9	69.5	10.3	InGaP matrix	[14]
InAs/GaAs	20	/	0.77	15.07	77	9.3	AlGaAs barrier	[104]
InAs/GaAs	20	2.3 × 10^10^	0.83	8.19	69.04	4.69	AlAs CL	[52]
InGaAs/GaAs	10	/	0.834	17.8	81.7	12.2	InGaAs CL	[56]
GaSb/GaAs	5	/	0.89	18.6	79	13.01	n-type doping	[78]
InAs/GaAs	10	1.8 × 10^10^	0.84	24.1	78	15.9	Control of GaAs layer thickness and annealing temperature	[105]
InAs/GaAs	5	4 × 10^10^	0.90	26.0	80	18.7	MOCVD	[94]
InAs/GaAs	20	/	0.926	12.58	83.63	9.75	QDs in the base region	[106]

As we can see in Table 1, the high *J*_sc_ could be obtained via Si doping and an increase in *V*_oc_ could be achieved by the use of wide bandgap matrix or CL, but plenty of researches show that high *J*_sc_ and *V*_oc_ are hard to realize at the same time. The low efficiency of the QD-IBSC can be mainly attributed to it that the QDs with the high density, good size homogeneity, and without defects are difficult to grow. As can be seen from Table 1, high-quality QDs obtained through controlling the annealing temperature greatly improve the efficiency. So the research on QD-IBSC should focus on the growth of high quality QDs in the future. Recently a new way to realize the IBSC with colloidal QDs has been developed. Colloidal QD-IBSC has the advantages of a high density of QDs, easy control of the QD size and fabrication, which may be a more promising way to solve this problem [27, 93]

To achieve high conversion efficiency, every factor should be considered and weighed. At present, the highest efficiency of InAs/GaAs reaches 18.7% (1 sun). The high-density QD solar cell with 5 layers stacked is grown by metalorganic chemical vapor deposition which suppresses the degradation of *V*_oc_, and a dual-layer anti-reflection coating is used to enhance the light absorption [94]. Therefore, in addition to increasing the number of QDs, light trapping is another way to improve the efficiency through enhancing light absorption. The main technologies used are different trapping structures, which reduce surface light reflection and increase light scattering. The methods commonly used are textures and back reflectors of different shapes, like planar back reflectors, pyramidal back reflectors. All of them show enhanced photocurrent compared with those without back reflectors [95–98].

## Conclusions

In this paper, we have reviewed the progress in In(Ga)As QD-IBSC. A large number of technologies have been used to solve the problems hindering the realization of efficient two-photon absorption with the *V*_oc_ preserved, including Si doping, cap layer, size control, strain compensation, using the matrix with large bandgap and type-II QDs. Nevertheless, till now, the QD-IBSC with higher energy conversion efficiency than the single-junction solar cell has not been reported yet and plenty of researches show that the high *J*_sc_ and *V*_oc_ are hard to be realized at the same time. To get a high *J*_sc_, multiple layers of QDs need to be grown to increase light absorption, but this in turn causes strain accumulate and voltage drop, which is due to the reason that the QDs with high density, no defects and good size uniformity are difficult to grow. Besides, the low efficiency of the cell is also related to the weak photoabsorption. In the future, researches on In(Ga)As QD-IBSC are supposed to focus on the growth of QD with high quality as well as light trapping technology. The summarized experimental results here may enlighten the further development of the In(Ga)As QD-IBSC.

## Data Availability

The datasets used and/or analyzed during the current study are available from the corresponding author on reasonable request.
